# Microbiome of Zoophytophagous Biological Control Agent *Nesidiocoris tenuis*

**DOI:** 10.1007/s00248-023-02290-y

**Published:** 2023-09-02

**Authors:** Yuta Owashi, Toma Minami, Taisei Kikuchi, Akemi Yoshida, Ryohei Nakano, Daisuke Kageyama, Tetsuya Adachi-Hagimori

**Affiliations:** 1 Institute of Agrobiological Sciences, National Agriculture and Food Research Organization (NARO), Tsukuba, Ibaraki, Japan; 2 Laboratory of Applied Entomology, University of Miyazaki, Miyazaki, Japan; 3 Frontier Science Research Center, University of Miyazaki, Miyazaki, Japan; 4 Department of Integrated Biosciences, Graduate School of Frontier Sciences, The University of Tokyo, Tokyo, Japan; 5 Shizuoka Prefectural Research Institute of Agriculture and Forestry, Shizuoka, Japan

**Keywords:** Symbiotic bacteria, Biological control, *Rickettsia*, *Wolbachia*, *Spiroplasma*, Mirid

## Abstract

Many insects are associated with endosymbionts that influence the feeding, reproduction, and distribution of their hosts. Although the small green mirid, *Nesidiocoris tenuis* (Reuter) (Hemiptera: Miridae), a zoophytophagous predator that feeds on plants as well as arthropods, is a globally important biological control agent, its microbiome has not been sufficiently studied. In the present study, we assessed the microbiome variation in 96 *N. tenuis* individuals from 14 locations throughout Japan, based on amplicon sequencing of the 16S ribosomal RNA gene. Nine major bacteria associated with *N. tenuis* were identified: *Rickettsia*, two strains of *Wolbachia*, *Spiroplasma*, *Providencia*, *Serratia*, *Pseudochrobactrum*, *Lactococcus*, and *Stenotrophomonas*. Additionally, a diagnostic PCR analysis for three typical insect reproductive manipulators, *Rickettsia*, *Wolbachia*, and *Spiroplasma*, was performed on a larger sample size (*n* = 360) of *N. tenuis* individuals; the most prevalent symbiont was *Rickettsia* (69.7%), followed by *Wolbachia* (39.2%) and *Spiroplasma* (6.1%). Although some symbionts were co-infected, their prevalence did not exhibit any specific tendency, such as a high frequency in specific infection combinations. The infection frequency of *Rickettsia* was significantly correlated with latitude and temperature, while that of *Wolbachia* and *Spiroplasma* was significantly correlated with host plants. The predominance of these bacteria and the absence of obligate symbionts suggested that the *N. tenuis* microbiome is typical for predatory arthropods rather than sap-feeding insects. *Rickettsia* and *Wolbachia* were vertically transmitted rather than horizontally transmitted from the prey. The functional validation of each symbiont would be warranted to develop *N. tenuis* as a biological control agent.

**Supplementary Information:**

The online version contains supplementary material available at 10.1007/s00248-023-02290-y.

## Introduction

Many insect species are closely associated with multiple endosymbionts that can affect the feeding, reproduction, and distribution of their hosts. Some of these associations have borne dependencies between the host and symbiont. For example, some herbivorous insects rely exclusively on nitrogen-poor substrates and require their symbionts for nutritional compensation [1], such as aphids with *Buchnera* [2], whiteflies with *Portiera* [3], and psyllids with *Carsonella* [4]. Furthermore, some blood-feeding insects have similar associations, such as tsetse flies with *Wigglesworthia* [5] and bedbugs with *Wolbachia* [6]. Such nutritional endosymbionts contribute significantly to the diversification of insect diets. Some endosymbionts have non-trophic effects on their hosts. *Wolbachia*, *Rickettsia*, *Spiroplasma*, and *Cardinium* induce reproductive phenotypes, such as cytoplasmic incompatibility (CI), male killing (MK), parthenogenesis induction (PI), and feminization (Fem), which are considered to be “selfish strategies” for the endosymbionts [7–12]. Some other endosymbionts are known to contribute to improving host fitness by increasing reproduction and development [13], conferring tolerance to thermal stress [14], and conferring resistance to pathogens [15].

Symbiotic bacteria often co-infect an individual in the same host population and show considerable variation in their infection patterns [16, 17]. The main factors that shape such patterns and symbiont community structure include host species [18], host plant [19], and geography [17]. For example, *Macrolophus* (Hemiptera: Miridae) are known to harbor one strain of *Wolbachia* and two species of *Rickettsia* (relatives of *Rickettsia bellii* and *Rickettsia limoniae*). *Macrolophus pygmaeus* harbors all these symbionts, whereas *Macrolophus melanotoma* (syn. *Macrolophus caliginosus*) harbors only *Wolbachia* and *R. limoniae* [18, 20, 21]. Although *M. pygmaeus* populations are geographically separated, their microbiomes are homogeneous, whereas the microbiomes of *M. melanotoma* are diverse [18, 21]. To fully understand the ecology and evolution of such species, it is important to understand how this variation in the symbiotic microbiota is involved in host adaptation. This is especially important given the potential role that many predatory insects play as biological control agents.

The small green mirid, *Nesidiocoris tenuis* (Hemiptera: Miridae), is a cosmopolitan species commonly used in the control of agricultural pests [22, 23]. They are zoophytophagous, which allows them to survive by feeding not only on arthropods but also on plants, which can augment their biological control activities but can also cause damage to crops [23–25]. *N. tenuis* are often found on *Sesamum indicum* (sesame) and *Cleome hassleriana* (cleome) in warm regions of Japan [23, 25]. In *N. tenuis*, two genera of symbionts, *Wolbachia* and *Rickettsia*, have been detected in Israeli populations and commercially available strains [26, 27]. Of these, the infection frequency of *Rickettsia* was found to be high (93–100%) in Israeli populations [26], whereas the infection frequency of *Wolbachia* remains unknown. Caspi-Fluger et al. [26] suggested that *Rickettsia* plays a nutritional role in zoophytophagous *N. tenuis* due to its high prevalence and abundance in adults and localization in the gut.

The aim of the present study was to elucidate the population structure of *N. tenuis* in Japan in terms of microbiome composition. We revealed the diversity of the microbiome in *N. tenuis* by 16S rRNA amplicon sequencing and diagnostic PCR assay. We also investigated whether the infection frequencies of *Wolbachia*, *Rickettsia*, and *Spiroplasma* were correlated with geography, climate, host plant, and host sex. These results reveal the complex relationships between *N. tenuis* and its symbionts, which may potentially contribute to improve the use of this species as a biological control agent.

## Materials and Methods

### Insect Collection

In total, 360 wild-caught adults of *N. tenuis* were collected from *Sesamum indicum* (sesame) or *Cleome hassleriana* (cleome) from 15 farms in Japan between 2017 and 2021 (Table S1). All individuals were stored in 99.5% ethanol at − 80 °C until DNA extraction was performed.

### DNA Extraction

The 360 DNA samples were extracted from the whole insect bodies using the Wizard® Genomic DNA Purification Kit (Promega Corporation, Madison, WI, USA) according to the manufacturer’s protocol. DNA was dissolved in 100 μL of Tris-EDTA (pH 8.0) and stored at − 30 °C until use.

### Amplicon Sequencing

For the selected 96 samples (Table S1), hypervariable V3/V4 regions of the 16S rRNA gene were amplified using the KAPA HiFi HotStart ReadyMix (Kapa Biosystems Inc., Wilmington, MA, USA) with V3V4_F primer (5′-TCGTCGGCAGCGTCAGATGTGTATAAGAGACAGCCTACGGGNGGCWGCAG-3′) and V3V4_R primer (5′-GTCTCGTGGGCTCGGAGATGTGTATAAGAGACAGGACTACHVGGGTATCTAATCC-3′). The reactions were initiated by denaturation at 95 °C for 3 min, followed by 25 cycles of 30 s at 95 °C, 30 s at 55 °C, 30 s at 72 °C, and a final extension step of 5 min at 72 °C. After purification of the PCR products using AMPure XP beads (Beckman Coulter Inc., Brea, CA, USA), eight cycles of a second PCR were performed to add barcode sequences to each product using the TG Nextera XT Index Kit v2 Set A (Illumina Inc., San Diego, CA, USA). All barcoded amplicons were pooled in equal concentrations and sequenced on the Illumina MiSeq platform using the MiSeq Reagent Nano Kit v3 (600 cycles) according to the manufacturer’s recommended protocol (https://icom.illumina.com/) to produce 300-bp paired-end reads.

### Raw Sequencing Data Analysis

The Illumina sequence data were processed using QIIME2 ver. qiime2-2020.11 [28]. The Illumina reads were demultiplexed based on the barcode sequences using “qiime demux emp-paired.” Denoizing and clustering were performed to obtain representative sequences and the feature table using “qiime dada2 denoise-paired” command. Taxonomic assignment to the representative sequences was then performed using “qiime feature-classifier classify-blast.” Sequences not identified as bacteria and all features with an abundance of < 0.01% were filtered out for further analysis. Data visualization was performed using the “qiime metadata tabulate” command, and the “qiime taxa barplot” command was used to generate a taxonomic bar plot. Alpha- and beta-diversity analyses were performed using the “qiime diversity alpha-rarefaction” and “qiime diversity core-metrics-phylogenetic” commands.

### Diagnostic PCR for Rickettsia, Wolbachia, and Spiroplasma

Diagnostic PCR for the insect reproductive manipulators *Rickettsia*, *Wolbachia*, and *Spiroplasma* was performed on 360 *N. tenuis* individuals from 15 farms in Japan (Table S1). PCR was performed using the Go Taq Green Master Mix (Promega) with 528-F (5′-ACTAATCTAGAGTGTAGTAGGGGATGATGG-3′) and 1044-R (5′-GTTTTCTTATAGTTCCTGGCATTACCC-3′) for *Rickettsia* [29], wsp81F (5′- TGGTCCAATAAGTGATGAAGAAAC-3′) and wsp691R (5′- AAAAATTAAACGCTACTCCA-3′) for *Wolbachia* [30], and Spiro_Nt_124F (5′-GACGGTACCTTACCAGAAAG-3′) and Spiro_Nt_409R (5′-TTCGTGCCTAAACGTCAGTG-3′) for *Spiroplasma* in *N. tenuis*. Reactions were initiated by denaturation at 95 °C for 3 min, followed by 35 cycles of 30 s at 95 °C, 30 s at 60 °C (for 528-F/1044-R) or 55 °C (for wsp81F/wsp691R) or 56 °C (for Spiro_Nt_124F/Spiro_Nt_409R), 60 s at 72 °C, and a final extension step of 10 min at 72 °C. DNA was detected by electrophoresis on a 2% agarose gel prestained with Midori Green Xtra (Nippon Genetics Co., Ltd., Tokyo, Japan) in Tris-acetate-EDTA buffer.

### Molecular Phylogenetic Analysis

Partial 16S rRNA sequences of *Wolbachia*, *Rickettsia*, and *Spiroplasma* isolated through amplicon sequencing were used for phylogenetic analyses. The datasets were registered in DDBJ (accession numbers: LC769520–LC769523). Phylogenetic trees based on the nucleotide sequences were constructed using the maximum likelihood method in MEGA 7.0 [31]. Kimura’s two-parameter model, evaluated with the best-fit method, was applied for the calculation [32].

### Statistical Analysis

The PCR-based presence or absence of each bacterium within a mirid individual was analyzed based on a generalized linear model (GLM) with a binomial distribution (with a logit link function). Latitude, longitude, annual mean temperature, host plant, and sex were analyzed as explanatory variables. Data with unidentified sex were excluded from the analysis. Based on the GLM, an analysis of variance (ANOVA) was performed to evaluate the effects of each explanatory variable. Geographical and climatic factors that showed significant correlations with the GLM analysis were plotted to explicitly evaluate differential infection frequency. In addition, graphical visualization and Fisher’s exact test for the presence/absence of each bacterium were performed for each host plant. Geographical data were obtained from the Geospatial Information Authority of Japan (https://maps.gsi.go.jp), and climatic data were obtained from the Automated Meteorological Data Acquisition System administered by the Japan Meteorological Agency (https://www.jma.go.jp). Co-infection of *Rickettsia*, *Wolbachia*, and *Spiroplasma* was analyzed using an association screening approach as previously described [33]. The *envelope* function from the *boot* package in R software was used to estimate the 95% confidence envelope for the distribution profile of the combination counts, simultaneously including all infection patterns. A global test based on the 95% confidence envelope was then performed. All of the above analyses were performed using R version 4.2.2 [34].

### Vertical Transmission Analysis

An isofemale line, K11, co-infected with *Wolbachia* and *Rickettsia*, was established from a female collected from population no. 1 in 2022 (Table S1). In the laboratory, K11 was reared using *Ephestia kuehniella* eggs (purchased in a frozen state from Agrisect Inc., Ibaraki, Japan) as the food source and *Crassula ovata* leaves as the oviposition substrate. The founder female and one of her G2 generation offspring were subjected to DNA extraction and amplicon sequencing as described above. All breeding was performed at 25 ± 1 °C with a light:dark regime of 14:10 h.

## Results

### Microbiomes of *N. tenuis* Inferred from 16S rRNA Gene Amplicon Sequencing

**Fig. 1:**
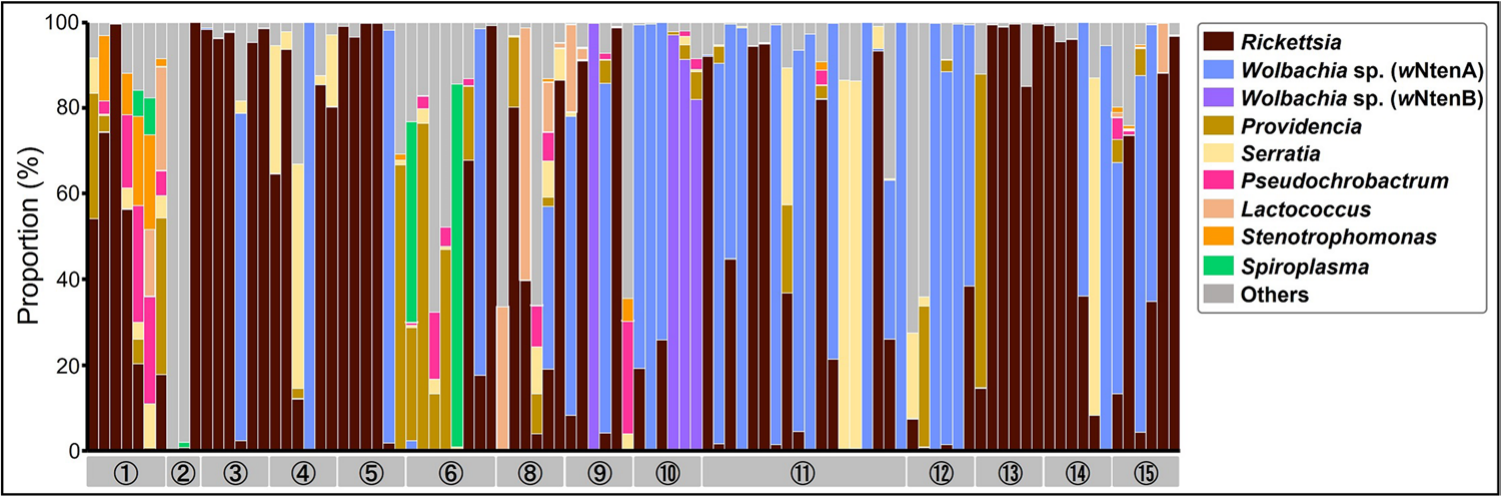
Proportion of bacterial sequences in 96 *N. tenuis* individuals collected from 14 regions in Japan. Sequences were obtained by amplicon sequencing of the hypervariable V3/V4 region of 16S rRNA. Assigned bacterial taxa are color coded as shown in the box on the right. Sequences with less than 25,000 total reads or 2000 reads per observed sample are categorized as “others.” The numbers at the bottom represent the geographic populations shown in Table S1

For the 96 *N. tenuis* individuals, the microbiomes were analyzed by amplicon sequencing of the hypervariable V3/V4 region of 16S rRNA, and a total of 4,625,099 reads were clustered into 77 operational taxonomic units (OTUs). The nine major OTUs (> 25,000 total reads and > 2000 reads per observed sample) were *Rickettsia* sp., two strains of *Wolbachia* sp., *Providencia* sp., *Serratia marcescens*, *Pseudochrobactrum* sp., *Lactococcus lactis*, *Stenotrophomonas* sp., and *Spiroplasma* sp., in order of frequency (Fig. 1, Table S2). Assuming that a mirid individual has the bacterium when it represented more than 1% of the tags analyzed, 69 out of 96 individuals had *Rickettsia* (71.9%), 30 individuals had *Wolbachia* sp. A (*w*NtenA, 31.3%), 4 individuals had *Wolbachia* sp. B (*w*NtenB, 4.2%), 25 individuals had *Providencia* (26.0%), 26 individuals had *Serratia marcescens* (27.1%), 18 individuals had *Pseudochrobactrum* (18.8%), 10 individuals had *Lactococcus lactis* (10.4%), 9 individuals had *Stenotrophomonas* (9.4%), and 5 individuals had *Spiroplasma* (5.2%).

### Infection Status of Rickettsia, Wolbachia, and Spiroplasma Inferred from Diagnostic PCR

**Fig. 2:**
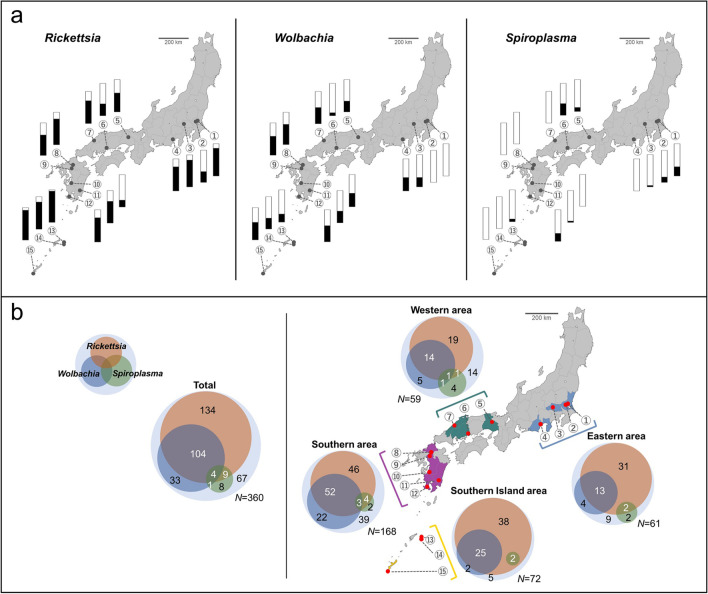
Infection frequencies of *Rickettsia*, *Wolbachia*, and *Spiroplasma* in each population of *N. tenuis* based on the diagnostic PCR assay. **a** Infection frequencies of *Rickettsia* (left panel), *Wolbachia* (center panel), and *Spiroplasma* (right panel). The frequencies of positive (black) and negative (white) individuals are shown with bar graphs. **b** Venn diagrams illustrating the co-infection status of *Rickettsia*, *Wolbachia*, and *Spiroplasma*. Each inner circle indicates the number of *N. tenuis* individuals infected with *Rickettsia* (red), *Wolbachia* (blue), and *Spiroplasma* (green)*.* Overlapping circles indicate multiple infections. The outer circles represent the total number of *N. tenuis* individuals that were examined. Population numbers correspond to those in Table S1

We further investigated the prevalence of *Rickettsia*, *Wolbachia*, and *Spiroplasma* in 360 individuals from 15 populations of *N. tenuis*. We found that 293 of the 360 individuals (81.4%) were infected with at least one bacterium. *Rickettsia* was the most prevalent being detected in 251/360 individuals (69.7%), followed by *Wolbachia* in 142/360 individuals (39.4%) and *Spiroplasma* in 22/360 individuals (6.1%), and the frequency of infection varied between populations (Fig. 2a, 2b; Table S3). Some *N. tenuis* were co-infected with multiple bacteria; 104 individuals were doubly infected with *Rickettsia* and *Wolbachia*, 9 individuals were doubly infected with *Rickettsia* and *Spiroplasma*, 1 individual was doubly infected with *Wolbachia* and *Spiroplasma*, and 4 individuals were triply infected (Fig. 2b).

### Correlation of Rickettsia, Wolbachia, and Spiroplasma with Latitude, Temperature, and Host Plants

**Fig. 3:**
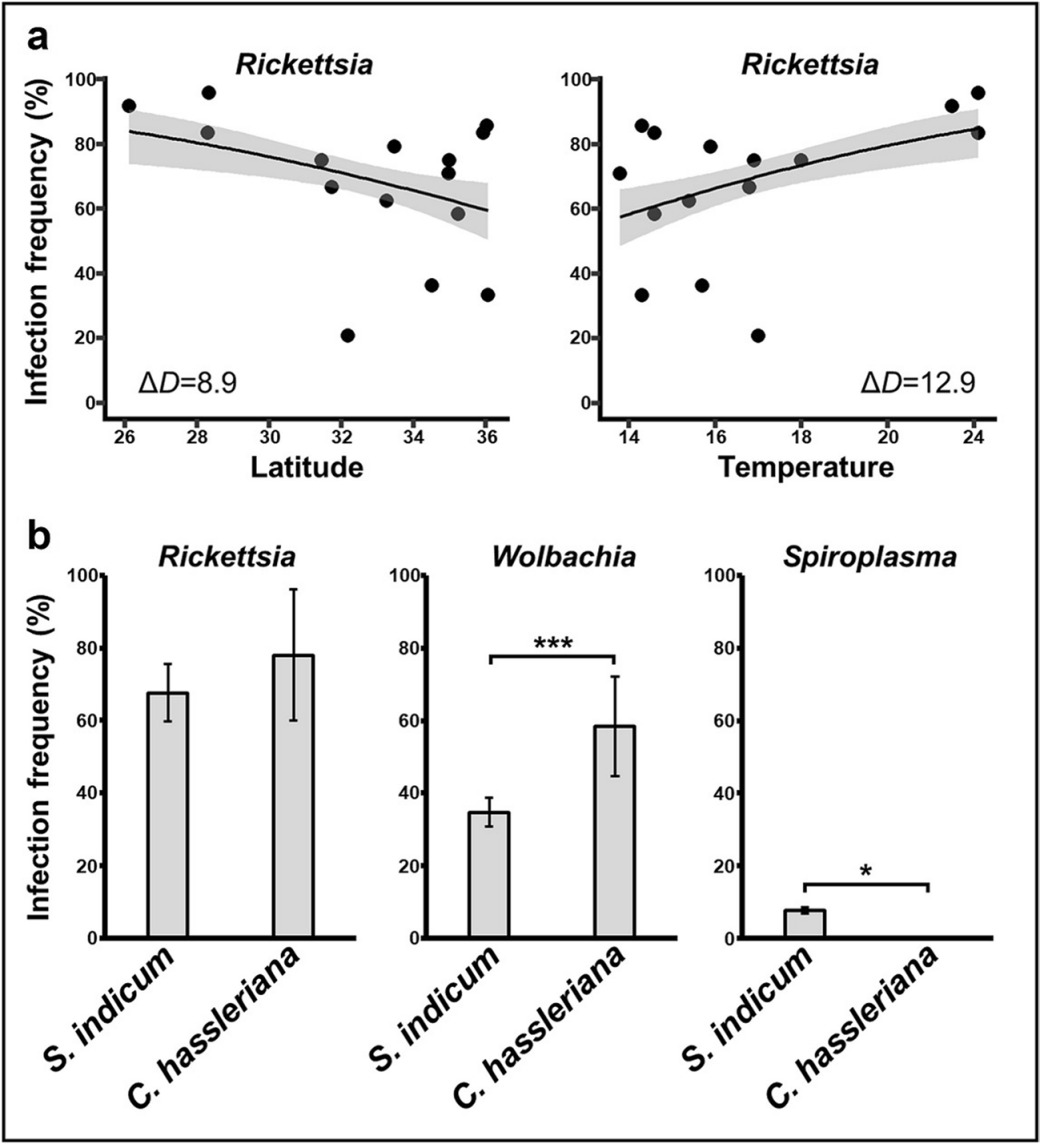
Relationship between infection frequencies of each symbiont (*Rickettsia*, *Wolbachia*, or *Spiroplasma*) in *N. tenuis* and each variable (latitude, temperature, or host plant). **a** A generalized linear model (GLM) with binomial error and logit-link function was plotted to estimate the effects of the correlation between *Rickettsia* and latitude or annual mean temperature for those significant differences detected (Table 1). The difference in deviance between the null hypothesis and the estimated model explained by each GLM is shown as Δ*D*, and the 95% confidence intervals are shaded in gray. **b** Infection frequencies of each endosymbiont in the host plants *Sesamum indicum* (sesame) and *Cleome hassleriana* (cleome). Error bars indicate 95% bootstrap percentiles (10,000 replicates). Asterisks indicate significant differences based on Fisher’s exact test (*P < 0.05; ***P < 0.0005)

GLMs showed that the infection frequency of *Rickettsia* was significantly correlated with latitude and annual mean temperature (Table 1; Fig. 2a). Regression analyses showed a higher frequency of *Rickettsia* at lower latitude and higher temperature (Fig. 3a). Furthermore, GLMs indicated that the host plant significantly affected the infection frequency of *Wolbachia* and *Spiroplasma* (Table 1). *Wolbachia* infection frequency was significantly higher on *C. hassleriana* (cleome) than on *S. indicum* (sesame), while *Spiroplasma* was not found on *C. hassleriana* (Fig. 3b). The association screening approach showed that no significant association was detected between the co-infection status of *Rickettsia*, *Wolbachia*, or *Spiroplasma* from 360 individuals of *N. tenuis*, and this was also the case when the analysis was run by area (Table 2).

**Table 1:** Correlation between geographic, climatic, and host factors and endosymbiont infections in natural populations of *N. tenuis* in Japan. The generalized linear model (GLM) incorporated the effects of geographic (latitude and longitude), climatic (average of annual temperature), host plant species, and sex variables of insects on the presence/absence of each endosymbiont with binomial error and logit-link function. Based on the GLM, an ANOVA was performed for each endosymbiont to estimate the *P*-value for each explanatory variable using chi-squared tests

	*Rickettsia*		*Wolbachia*		*Spiroplasma*	
Variable	df	*P*-value	df	*P*-value	df	*P*-value
Latitude	1	0.0027*	1	0.3649	1	0.1621
Longitude	1	0.6407	1	0.0059	1	0.1762
Temperature	1	0.0003*	1	0.9960	1	0.3613
Host plant	1	0.0889	1	0.0002*	1	0.0014*
Sex	1	0.0680	1	0.1053	1	0.1112

*Significant *P*-values (< 0.05) after Bonferroni correction

**Table 2:** Co-infection status for *Rickettsia*, *Wolbachia*, and *Spiroplasma* of *N. tenuis* by area as seen through association screening analysis. The number of data points is shown as *N*, and the *P*-value from the global test based on the 95% confidence envelope is shown as *P*

Area	*N*	*P*
Total	360	0.1092
Eastern area	61	0.3268
Western area	59	0.2164
Southern area	168	0.2352
Southern Island area	72	0.6096

### Molecular Phylogenetic Analysis of Rickettsia, Wolbachia, and Spiroplasma

**Fig. 4:**
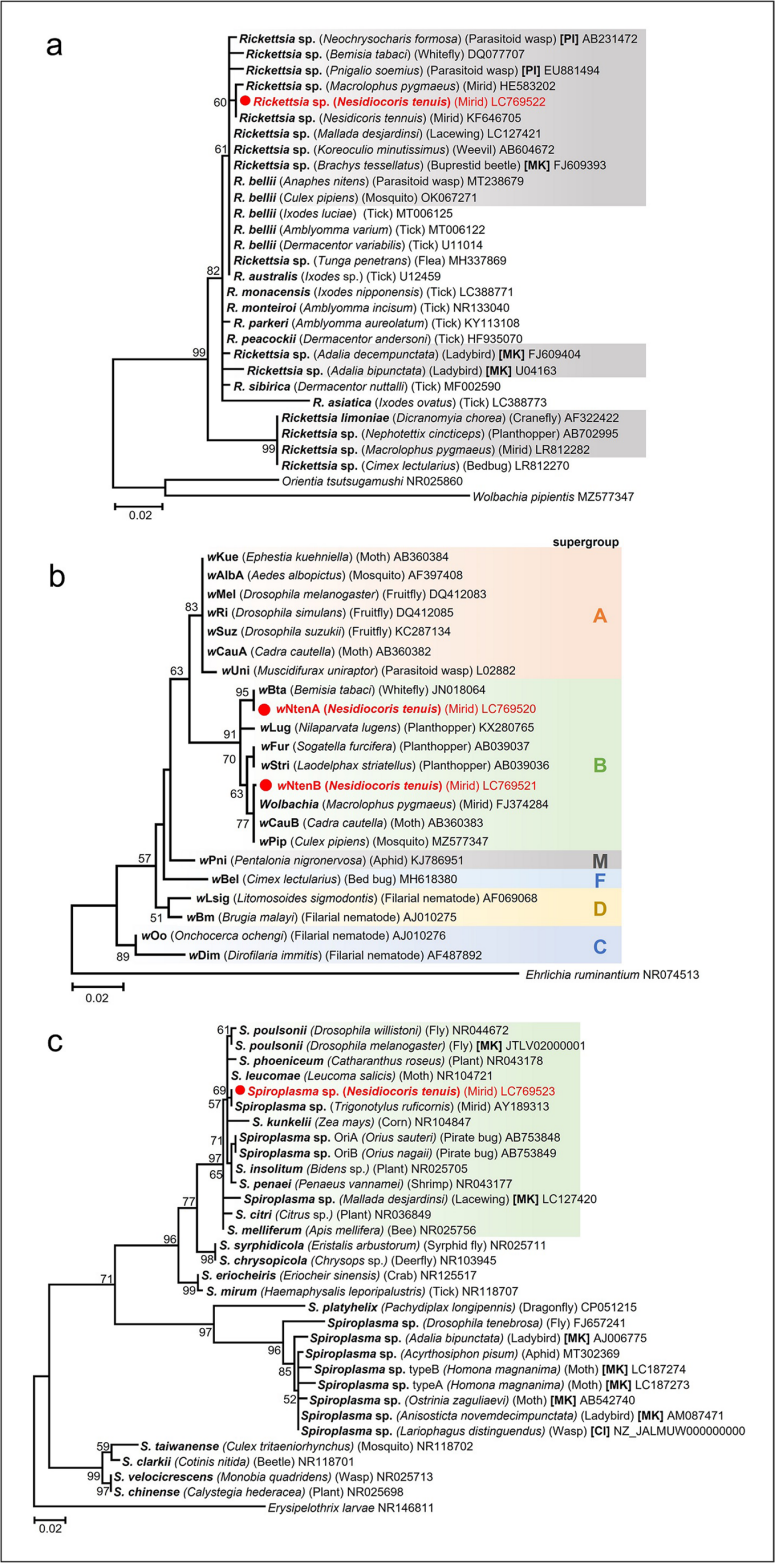
Phylogenetic trees based on the 16S rRNA gene sequences of *Rickettsia*, *Wolbachia*, and *Spiroplasma*. These trees were generated using the maximum likelihood method based on the Kimura 2-parameter model [32] with 1000 bootstrap replicates. Bootstrap values < 50% are not shown. The symbionts from *N. tenuis* are shown in red. The host organisms are given in parentheses, whereas the accession number is provided after each OTU. The scale bar indicates 0.02 substitutions per site. **a** Phylogenetic tree of *Rickettsia* based on 360 nucleotide sites. MK and PI represent *Rickettsia* isolates that cause male killing and parthenogenesis induction, respectively. The OTUs from insect symbionts are shaded gray. The outgroups are *Wolbachia pipientis* and *Orientia tsutsugamushi*. **b** Phylogenetic tree of *Wolbachia* based on 360 nucleotide sites. *Wolbachia* supergroups are depicted on the right side. The outgroup is *Ehrlichia ruminantium*. **c** Phylogenetic tree of *Spiroplasma* based on 384 nucleotide sites. MK and CI represent *Spiroplasma* isolates that cause male killing and cytoplasmic incompatibility, respectively. The Citri-Poulsonii clade is shaded green. The outgroup is *Erysipelothrix larvae*

To infer the phylogenetic position of *Rickettsia*, *Wolbachia*, and *Spiroplasma*, nucleotide sequences (360, 360, and 384 bp, respectively) obtained through amplicon sequencing were subjected to maximum-likelihood tree reconstruction. In the *Rickettsia* phylogeny, the *Rickettsia* in the *N. tenuis* from Japanese populations was identical to that from the Israeli population [26], which was closely related to *Rickettsia bellii* (Fig. 4a). Of the two *Wolbachia* isolates in *N. tenuis*, one is the major isolate (*w*NtenA), which was detected in 30 out of 96 individuals, and the other is the minor isolate (*w*NtenB), which was detected in 4 out of 96 individuals. In the *Wolbachia* phylogeny, *w*NtenA and *w*NtenB both belonged to the *Wolbachia* supergroup B (Fig. 4b). *w*NtenA was closely related to the *Wolbachia* from the whitefly *Bemisia tabaci*, and *w*NtenB was closely related to those from *Macrolophus pygmaeus*, *Cadra cautella*, and *Culex pipiens*. In the *Spiroplasma* phylogeny, the *Spiroplasma* in *N. tenuis* fell into the Citri-Poulsonii clade, a large group consisting of *S. citri*, *S. melliferum*, *S. kunkelli*, *S. penaei*, *S. insolitum*, *S. leucomae*, *S. phoeniceum*, and *S. poulsonii* (Fig. 4c).

### Vertical Transmission of Rickettsia and Wolbachia

A total of 35,102 reads were obtained from the amplicon sequence analysis of the founder female of strain K11. Two major OTUs were classified as *Rickettsia* (17,396 reads) and *Wolbachia* (17,642 reads), respectively (Fig. S1). In G2, a total of 35,397 reads were clustered into *Rickettsia* (11,158 reads) and *Wolbachia* (24,097 reads) (Fig. S1). These nucleotide sequences of *Rickettsia* and *Wolbachia* were identical to those of *Rickettsia* and *w*NtenA obtained from *N. tenuis* in Fig. 4, respectively.

## Discussion

This study demonstrated the high prevalence of *Rickettsia* and *Wolbachia* in Japanese *N. tenuis* populations (Fig. 1; Table S2), which is consistent with the results of a previous study on Israeli *N. tenuis* [26]. These symbionts induce reproductive phenotypes in insect hosts, and some of them can improve host fitness [9, 12, 13, 35]. Similarly, *Spiroplasma* manipulates host reproduction in some insects and can confer resistance to various parasites [10, 36, 37]. To the best of our knowledge, our study is the first to detect *Spiroplasma* in *N. tenuis.* In addition, *Providencia*, *Serratia marcescens*, *Pseudochrobactrum*, *Stenotrophomonas*, and *Lactococcus lactis* were found to be relatively abundant bacterial taxa in the *N. tenuis* population in Japan (Fig. 1; Table S2). *S. marcescens* and *Providencia* are commonly present in the environment [38, 39], and *S. marcescens* was also isolated from *N. tenuis* in a previous study [27]. Our study showed widespread infection of *S. marcescens* and *Providencia* among individuals but with low sequence reads per individual (Table S2), which may suggest opportunistic pathogenic properties of these bacteria [39, 40]. In the mosquito species *Aedes aegypti*, *S. marcescens* is present as a gut commensal bacterium that influences viral vector competence [41]. *Stenotrophomonas*, *Pseudochrobactrum*, and *Lactococcus lactis* have also been reported to be latent in the environment [42–44] and insect gut [45]. These results reveal a diversity of endosymbiotic microbes in natural populations of *N. tenuis*. The fact that none of the bacterial species found in this study were fixed in zoophytophagous *N. tenuis* suggests the absence of obligate symbionts in *N. tenuis*, a trait more typical for predatory arthropods rather than sap-feeding insects.

*Rickettsia*, *Wolbachia*, and *Spiroplasma* manipulate host reproduction in various insects [7–10, 12]. We found all possible combinations of these genera in *N. tenuis* individuals. Given that there was no correlation between the frequency of *Rickettsia*, *Wolbachia*, or *Spiroplasma* and the host sex (Table 1), it is unlikely that these symbionts induce MK, PI, or Fem in *N. tenuis*, which would otherwise result in a female-biased sex ratio and preferential presence of the symbiont in females. Coexisting symbionts may engage in interactions that are either negative or positive [46, 47]. Although no significant association with infection frequency was found (Table 2), further analysis of reproductive phenotypes or life history traits in various symbiont combinations is needed to understand the complex symbiotic system of *N. tenuis* populations and to propose the optimal biological control agent. *Rickettsia*, *Wolbachia*, or *Spiroplasma* have been detected in other carnivorous arthropods, such as mirids [18, 21, 26], coccinellids [48], and lacewings [7]. Feeding on other arthropods may have increased the chance of acquiring the symbionts common to prey species for *N. tenuis* [49].

The *Rickettsia* found in the present study is identical based on the partial sequence of the 16S rRNA gene to the *Rickettsia* sequence previously reported in *N. tenuis* [26], which is closely related to the *R. bellii* group. Previously, *Rickettsia* was detected in the gut lumen along the digestive tract of *N. tenuis*, while *Wolbachia* was detected in the surrounding epithelial cells [26]. A similar distribution of *Rickettsia* was elucidated in *Macrolophus*; both *R. bellii* and *R. limoniae* were found in the gut of *M. pygmaeus* and *M. melanotoma* [20, 21]. Although no correlation was found between the infection frequency of *Rickettsia* and the host plant, future studies should investigate the possible involvement of *Rickettsia* in the nutrient metabolism, including the zoophytophagous trait, of this species. It should be noted that no significant effects of *Rickettsia* and *Wolbachia* on the fitness traits of nymphal development and fecundity were detected in *M. pygmaeus* [18]. Although the Israeli populations harbored *Rickettsia* at a consistently high frequency (93–100%) [26], Japanese populations harbored *Rickettsia* at variable and relatively low frequencies (20.8–95.8%) (Fig. 2; Table S3). The fact that the high infection frequency of *Rickettsia* was associated with lower latitude and higher annual mean temperature (Fig. 3; Table 1) suggests the possibility that *Rickettsia* may provide positive effects to the host, such as heat tolerance, under high temperature [50] or negative effects under low temperature. *Rickettsia* infection is known to upregulate the expression of stress response genes in *B. tabaci*, which may underlie the mechanism of heat tolerance [50]. Furthermore, the supercooling point of *M. pygmaeus* exhibited a decrease upon the removal of its symbionts (two *Rickettsia* species and *Wolbachia*); however, it remains uncertain which bacterium influenced to the freezing susceptibility [51]. These possible effects of *Rickettsia* on hosts may explain the variable frequency of *Rickettsia* in Japanese populations of *N. tenuis*. Alternatively, *Rickettsia* may have no effect on host temperature sensitivity and our observation simply reflects the temperature sensitivity of *Rickettsia* itself [52].

Of the two supergroup B *Wolbachia* strains identified in this study, the major strain *w*NtenA was identical in terms of the partial 16S rRNA gene sequence to the *Wolbachia* strain found in *B. tabaci*. Despite the existence of a predator–prey relationship between *N. tenuis* and *B. tabaci* [23, 24], it is unlikely that the detected *Wolbachia* bacteria are exclusively derived from undigested *B. tabaci* remaining in the gut. This is because a large number of *Wolbachia* sequence reads were obtained using amplicon sequencing. Furthermore, vertical transmission was confirmed by breeding individuals under controlled laboratory conditions where they were not exposed to *B. tabaci* (Fig. S1). In *B. tabaci*, *Wolbachia* can be transmitted horizontally through plants and subsequently transmitted vertically to offspring [53]. The possibility that *N. tenuis* acquired *Wolbachia* from plants might be supported by the observed correlation between the frequency of *Wolbachia* and host plants.

The other strain, *w*NtenB, was identical with respect to the partial sequence of the 16S rRNA gene to the *Wolbachia* strain found in *M. pygmaeus*, which is known to induce strong CI [54]. Although strong CI is generally considered to cause widespread infection of the symbiont within the host population [55], *w*NtenB was rare (4 out of 96) in the *N. tenuis* populations in Japan. Furthermore, we did not observe co-infection of *w*NtenA and *w*NtenB, so whether they are in conflict or not remains unclear.

In the present study, we detected *Spiroplsma* from *N. tenuis* for the first time. *Spiroplasma* has also been detected in other hemipteran species, such as planthoppers, leafhoppers, and *Orius* predatory bugs [56–58]. In leafhoppers, *Spiroplasma* is transmitted horizontally between plants and insects [56]. Interestingly, we found that the infection frequency of *Spiroplasma* differed depending on the host plant (Fig. 3; Table 1), and the partial sequence of the 16S rRNA gene of *Spiroplasma* from *N. tenuis* was related to that of another mirid bug from Taiwan, *Trigonotylus ruficornis* (Fig. 4c). Future studies should aim to directly test whether *Spiroplasma* can be horizontally transmitted via plants.

The presence of symbionts may have important implications for the practical use of the predators as biological control agents. In particular, the high infection frequency of *Rickettsia* and *Wolbachia* may indicate their ability to manipulate host reproduction or their positive effects on host fitness. Significant differences in infection rates among host plants and geographic regions may affect the effectiveness of its use as a biological control agent, including its choice of insectary plants and its ability to propagate in the regions where it is used, both of which remain unexplored. Our findings highlight the potential importance of these symbionts, which may strongly affect the intrinsic rate of increase and confound the population dynamics of *N. tenuis*. We encourage future studies to determine the impact of each symbiont on this important biological control agent.

### Supplementary information

ESM 1 (PDF 231 kb)

ESM 2 (XLSX 72 kb)

## Data Availability

The datasets presented in this study can be found in online repositories. Repository names and accession numbers can be found at https://www.ddbj.nig.ac.jp/, DRR480549–DRR480644, LC769520–LC769523.
