# Supplementary material for: Microbiome of Zoophytophagous Biological Control Agent Nesidiocoris tenuis
Source: Microb Ecol. 2023 Sep 2;86(4):2923–33. doi: 10.1007/s00248-023-02290-y (PMC10640431; doi:10.1007/s00248-023-02290-y)
Supplement: Supplementary file 1 — (PDF 231 kb) [file 248_2023_2290_MOESM1_ESM.pdf]

Microbial Ecology

Microbiome of zoophytophagous biological control agent *Nesidiocoris tenuis*

Yuta Owashi,[1] Toma Minami,[2] Taisei Kikuchi,[3,4] Akemi Yoshida,[3] Ryohei Nakano,[2,5] Daisuke Kageyama,[1]# Tetsuya Adachi-Hagimori[2]#

[1] Institute of Agrobiological Sciences, National Agriculture and Food Research Organization (NARO), Tsukuba, Ibaraki, Japan
[2] Laboratory of Applied Entomology, University of Miyazaki, Miyazaki, Japan
[3] Frontier Science Research Center, University of Miyazaki, Miyazaki, Japan
[4] Department of Integrated Biosciences, Graduate School of Frontier Sciences, The University of Tokyo, Tokyo, Japan
[5] Shizuoka Prefectural Research Institute of Agriculture and Forestry, Shizuoka, Japan

#Address correspondence to Tetsuya Adachi-Hagimori, tadachi@cc.miyazaki-u.ac.jp
#Address correspondence to Daisuke Kageyama, kagyamad@affrc.go.jp

Yuta Owashi and Toma Minami contributed equally to this work.

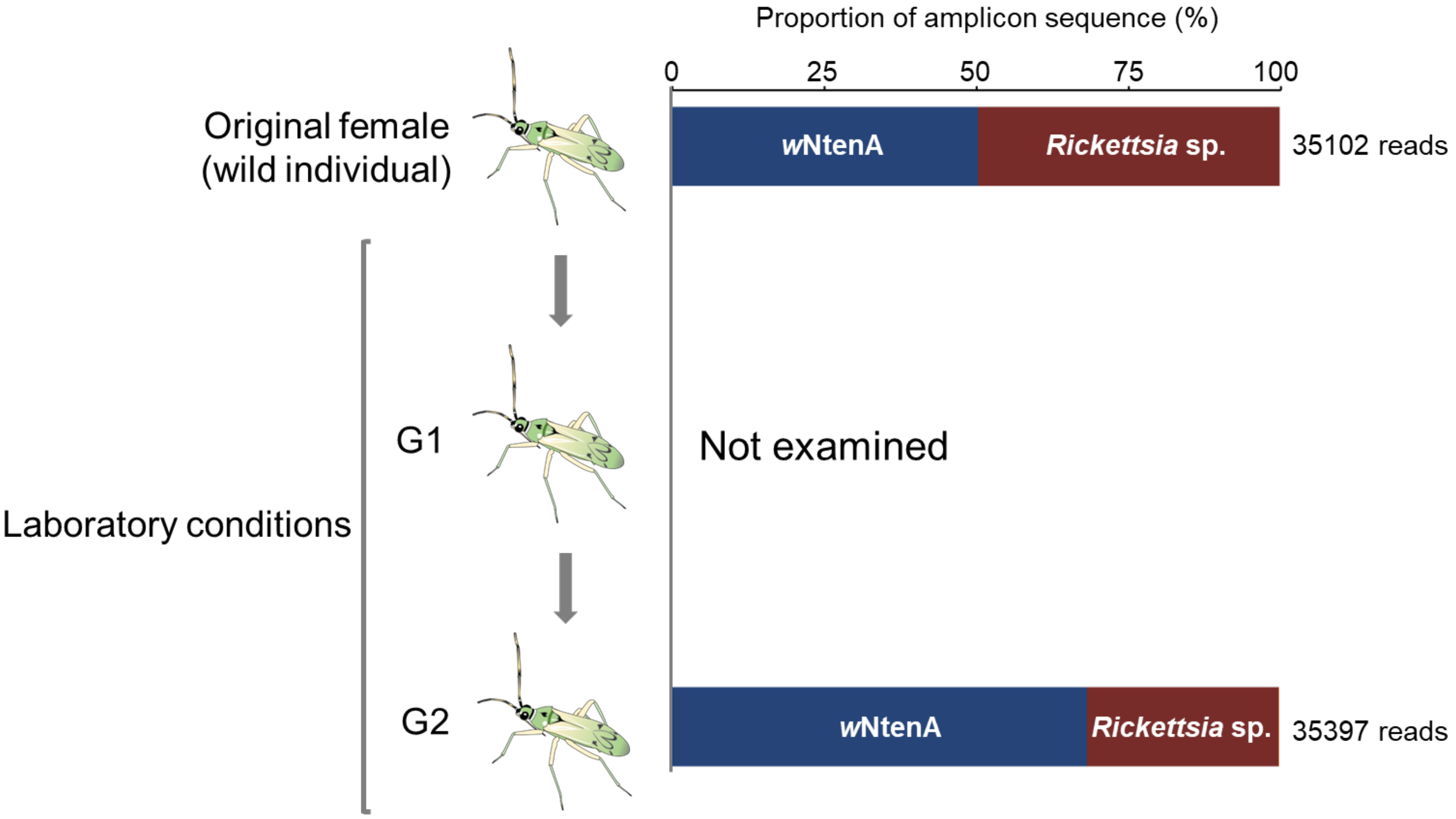

Fig. S1 Results of amplicon sequencing of the hypervariable V3/V4 region in 16S rRNA of the laboratory strain of *N. tenuis* derived from population No. 1 in Table S1. Assigned bacterial taxa are color-coded as blue for *w*NtenA and red for *Rickettsia*, consistent with Fig. 1. The next generation of an original female represents G1 and that of G1 represents G2. Under laboratory conditions, eggs of *Ephestia kuehniella* and leaves of *Crassula ovata* were offered as food and oviposition substrate, respectively
